# Performance benchmarks for open source porous electrode theory models

**DOI:** 10.1016/j.heliyon.2024.e27830

**Published:** 2024-03-20

**Authors:** Surya Mitra Ayalasomayajula, Daniel Cogswell, Debbie Zhuang, R. Edwin García

**Affiliations:** a School of Material Engineering, Purdue University, West Lafayette, IN, 47906, United States of America; b Department of Chemical Engineering, Massachusetts Institute of Technology, Cambridge, MA, 02139, United States of America

## Abstract

The electrochemical response characteristics of existing and emerging porous electrode theory (PET) models was benchmarked to establish a common basis to assess their physical reaches, limitations, and accuracy. Three open source PET models: dualfoil, MPET, and LIONSIMBA were compared to simulate the discharge of a LiMn_2_O_4_-graphite cell against experimental data. For C-rates below 2C, the simulated discharge voltage curves matched the experimental data within 4% deviation for dualfoil, MPET, and LIONSIMBA, while for C-rates above 3C, dualfoil and MPET show smaller deviations, within 5%, against experiments. The electrochemical profiles of all three codes exhibit significant qualitative differences, despite showing the same macroscopic voltage response, leading the user to different conclusions regarding the battery performance and possible degradation mechanisms of the analyzed system.

## 1 Introduction

Lithium-ion batteries, LIBs, are one of the cornerstone technologies of modern society for its ability to tailor high power and energy densities [1], [2], [3], [4], for applications ranging from automotive, grid storage, and aerospace applications to portable electronics [5], [6]. Increasing demand for LIBs has propelled research in areas such as material discovery, charging speeds, battery life, and operational safety. In this context, the use of numerical models has been key [7], [8], [9], [10], because of their ability to integrate and verify experiments, help identify new physics, and guide the design of LIBs.

The electrochemical, mechanical, and thermal processes that occur on a microscopic scale in a rechargeable battery directly impact the macroscopic performance of a LIB. Over the past three decades, a substantial effort has been dedicated to develop physics-based battery models to address challenges at different length scales, ranging from the atomic level to the battery pack level [8], [9], [10], [11], [12], [13].

Spatially resolved microscale models directly use experimental or computer-generated microstructural data without any homogenization approximations [14]. García et al. [15], [16] pioneered their use to quantify the spatial distribution of lithium, electrical potential, and mechanical stresses during cycling, and help improve microstructure design. Smith et al. [17] extended this approach [15], [16], and showed the impact of particle size distribution, particle packing, and morphology on the macroscopic behavior of the cell highlighting microstructural heterogeneities that lead to capacity loss for a LiCoO_2_-graphite cell. Chung et al. [18] studied the effect of particle size polydispersity in computer generated and experimental microstructures and showed that monodisperse particles deliver high power while polydisperse particles deliver high energies, thus provided a design criteria for battery microstructures. Müller et al. [19] developed a 3D model by starting from experimentally reconstructed microstructures to quantify the effects of inhomogeneities across multiple length scales to determine the quality of manufactured electrodes. Lu et al. [20] developed a 3D microstructurally resolved model, including the binder and proposed that graded microstructures improved the macroscopic performance at high C-rates.

Spatially resolved microstructural models provide useful insights to improve the microstructure and tune the overall battery performance, but they are computationally expensive [14]. In contrast, macroscopic models coarse grain average microscopic physical descriptions and use upscaling homogenization techniques [21], [22], [23], [24], [25], to capture the microscale physics and describe the macroscopic charge-discharge behavior of a LIB. In particular, porous electrode theory (PET) models, also referred to as pseudo-2D (p2D) models [26], [27], [28], [29], [30], were pioneered by Newman and Tiedemann [31], and were extended by Doyle et al. [32] to LIBs by introducing concepts of concentrated solution theory and intercalation kinetics [32], [33]. This approach decoupled the electrolyte porous phase from the active material granular phase to physically describe the coupled chemical and electrical fields across the thickness of the cathode, separator, and anode layers while describing lithium intercalation/deintercalation process in a representative spherical particle. Since then, the scope of these models has evolved to further include phase separation [34], [35], [36], mechanical [37], thermal [38], [39], and degradation effects [40], [41], [42] to further increase their complexity.

Open source implementations of porous electrode theory include releases such as, dualfoil [32], LIONSIMBA [39], and MPET [36]. Dualfoil is the most widely known code [43]. It is Fortran based and developed by Doyle as part of his doctoral thesis [44]. Arora et al. [41] introduced models to include capacity fade mechanisms, while Thomas et al. [45] introduced a thermal model and coupled it to the original dualfoil framework. Overall, the code is capable of solving time-dependent electrochemical charge-discharge cycles along with thermal effects and degradation mechanisms which are included as side reactions. One drawback of the code is that it needs the f77 Fortran compiler to run, and editing the source code to use a new solver or implement new mechanisms is difficult.

Recently, LIONSIMBA was developed as an open source MATLAB code by Torchio et al. [39]. This PET modular code also solves thermal effects and has been proven useful to design battery packs. LIONSIMBA does not include phase transition and degradation effects at the closing of this paper. Although the code is free and readily available, a purchased MATLAB license is required.

Most recently, the Multiphase Porous Electrode Theory, MPET, an open source Python code, was developed by Smith et al. [36]. It provides an implementation of porous electrode theory and incorporates phase transformation kinetics at the particle scale by solving the Cahn-Hillard equation. MPET only supports isothermal calculations and does not include degradation mechanisms.

The ability to modify open source software by the user, include new physical mechanisms, or automate the analysis to run numerous simulations has set the stage to perform data analytics on battery performance. However, in most of the available codes, documentation is at times limited, missing tests to assert the validity of the different components (or modules), or require lengthy editing processes to incorporate new chemistries. Specifically, tests for model validation are available on very few chemistries, and the used parameter values can significantly change from one PET model to another, which not only poses a steep learning curve for a new user but creates unnecessary confusion.

In this paper, we compare three porous electrode theory model implementations, namely dualfoil, [32], [43], MPET, [36] and LIONSIMBA, [39]. Their cell characteristics and electrochemical profiles are compared against the well-documented LiMn_2_O_4_-graphite cell experimental data^1^ as reported by Doyle et al. [46]. In order to provide a baseline framework to assess the performance and physical implications from selecting a model, the overall computational and electrochemical performance was assessed, thus defining a set of benchmarking tests that enable current and new users, existing and emerging developers, from both industry and academia, to select, develop, and advance, accurate models.

## 2 Theoretical framework

### 2.1 Generalities

**Figure 1:**
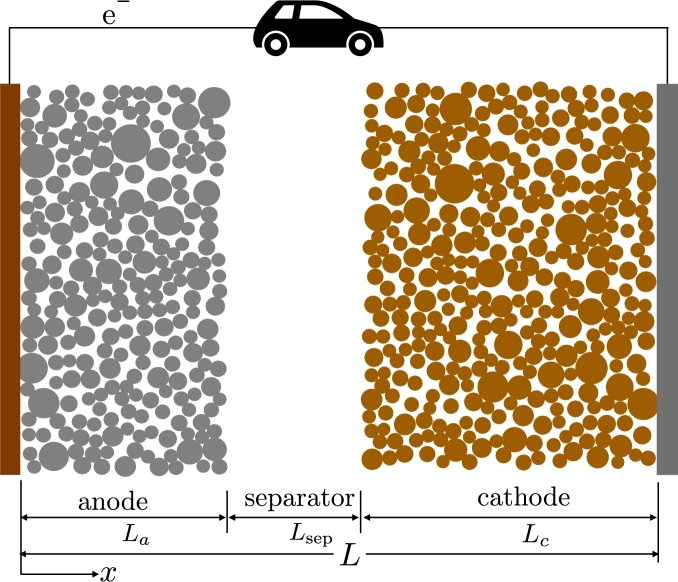
Schematic representation of a Li-ion cell microstructure. While the electrolyte is present in all three regions, the anode is typically conformed of graphite , and the cathode is a composite of ceramic particles such as . The particle shape and size distribution are determined by the chemistry and used powders, and specifies the macroscopic power and energy density of the cell, [47]. The separator is sandwiched between these two regions and is a porous but electronically insulating region. The charge and mass transport of lithium are typically solved along the through-thickness direction by developing a set of homogenized transport equations [48]. The microstructural richness is typically replaced by a set of averaged properties. See text for details.

LIBs are three-layer structures usually made up of two electrolyte-filled porous electrodes with an intervening separator, as shown in Fig. 1. During LIB discharge, the lithium stored in the particles of electrochemically active material in the anode is deintercalated into the electrolyte in the pores to then diffuse to the cathode layer across the separator and intercalate into the cathode active material. The corresponding electrons flow through the external circuit from the anode to the cathode. The process is reversed when an electric current is applied in the opposite direction.

### 2.2 Microstructural properties

Porous electrode theory models describe the coarse grained average mass and charge transport, where each layer is microstructurally described in terms of its porosity, *ϵ*, the volume fraction of the space left by the particles of active material in the electrode, defining a statistically representative longer path for diffusion. The macroscopic transport properties, $D_{e}^{\mathrm{eff}}=D_{e}^{\circ }\epsilon /\tau$, for mass diffusion, and $\sigma_{e}^{\mathrm{eff}}=\sigma_{e}^{\circ }\epsilon /\tau$, for charge transport, in the electrolyte phase are both coupled to the tortuosity, *τ*, of the electrode layer, through the Bruggeman relation, $\tau =1/\epsilon^{b}$, where *b* is the Bruggeman exponent [49], [50], [51]. Similarly, the reactive area density, $a=\frac{S(1-\epsilon )}{r_{p}}$, defines the surface area per unit volume of electrode particles in contact with the electrolyte, where *S* is the shape factor, a statistical measure of particle shape, distribution, and alignment, and $r_{p}$ is the average size of a representative electrode particle [18], [46], [52]. The list of used symbols is summarized in Table 1.

**Table 1:** List of symbols used in this paper.

Symbol	Description	Units
*a*	reactive area density of electrode	–
*b*	Bruggeman exponent of the electrode	–
*c*_*e*_	electrolyte phase concentration	mol/m^3^
$\overset{˜}{c_{e}}$	non-dimensionalized electrolyte phase concentration	–
*c*_*s*_	solid phase concentration	mol/m^3^
${\overset{¯}{c}}_{s}$	lithium concentration on electrode surface	mol/m^3^
$c_{s}^{\max}$	solubility limit of lithium in solid phase	mol/m^3^
$D_{e}^{\mathrm{eff}}$	macroscopic lithium ion diffusivity in the electrolyte phase of electrode	m^2^/s
$D_{e}^{\circ }$	lithium ion diffusivity in the electrolyte	m^2^/s
*D*_*s*_	lithium diffusivity in the solid phase of electrode	m^2^/s
*F*	Faraday's constant	C/mol
*I*_app_	applied current density under galvanostatic operation	A
*i*_*e*_	current density in electrolyte phase	A/m^2^
*i*_*s*_	current density in solid phase	A/m^2^
*i*_∘_	exchange current density	A/m^2^
*j*	reaction flux at the electrode surface	A/m^2^
*k*	charge transfer reaction rate constant	$\frac{m^{2.5}}{{\mathrm{mol}}^{0.5}\;s}$
*L*	thickness of the cell	μm
*L*_*a*_	thickness of the anode layer	μm
*L*_*c*_	thickness of the cathode layer	μm
*L*_sep_	thickness of the separator	μm
*R*	universal gas constant	J/mol.K
*r*_*p*_	average radius of a representative spherical particle in electrode	μm
*S*	shape factor of the electrode particles	–
*T*	absolute temperature	K
*t*	time	s
*t*_+_	lithium ion transference number	–
*U*	open circuit voltage of electrode active material	V
*x*	position along the thickness of the cell	μm
*α*	forward reaction transfer coefficient	–
*ϵ*	volume fraction of electrolyte in electrode	–
*η*_*s*_	surface overpotential	V
$\sigma_{e}^{\mathrm{eff}}$	macroscopic electrical conductivity of electrolyte phase in electrode	S/m
$\sigma_{e}^{\circ }$	electrical conductivity of electrolyte	S/m
$\sigma_{s}^{\mathrm{eff}}$	macroscopic electrical conductivity of solid phase in electrode	S/m
$\sigma_{s}^{\circ }$	electrical conductivity of solid phase of electrode	S/m
*τ*	tortuosity of the electrode microstructure	–
Φ	cell voltage	V
*ϕ*_*e*_	electrolyte phase electrical potential	V
*ϕ*_*s*_	solid phase electrical potential	V

### 2.3 Interfacial reactions

At the particle-electrolyte interface, lithium ions in the electrolyte, ${\mathrm{Li}}^{+}$, undergo a charge transfer reaction, ${\mathrm{Li}}^{+}+e^{-}⇌\mathrm{Li}$, thereby intercalating into the electrochemically active particle. The net flux of lithium is a sum of forward and backward reaction rates that are driven by the differences in free energy between the electrolyte and electrode particle surface [53], [54], [55]. The forward and backward reaction rates are empirically related to the exponential of driving force, and the lithium concentration in the electrolyte and electrode surface. When the electrochemical potential difference is the only driving force for intercalation, the lithium ion flux to and from the particle surface is traditionally modeled as a Butler-Volmer relation [32], [33], [56], [57]:

(1) $$\begin{array}{c} j=i_{\circ }\left[\exp\left(\frac{\alpha F\eta_{s}}{RT}\right)-\exp\left(-\frac{(1-\alpha )F\eta_{s}}{RT}\right)\right] \end{array}$$

Here, the exchange current density is, $i_{\circ }=Fkc_{e}^{\alpha }{\left(c_{s}^{\max}-{\overset{¯}{c}}_{s}\right)}^{\alpha }{\left({\overset{¯}{c}}_{s}\right)}^{\left(1-\alpha \right)}$, and the surface overpotential is, $\eta_{s}=\phi_{s}-\phi_{e}-U$.

### 2.4 Porous electrode theory

#### 2.4.1 Generalities

Each electrode layer is a non-homogeneous medium composed of an active material solid particle phase and an electrolyte phase. The description of the microscale transport equations for a homogeneous medium are volume averaged into a representative volume element, RVE, that describes the local state of both solid and electrolyte phases [31], [48], [58], *i.e.*, the porous electrode is treated as a superposition of the average solid and electrolyte phases, with coarse grained, averaged properties [32], [48].

Spatially resolved microscale mass and charge transport in the homogeneous medium, *i.e.*, the electrolyte or the solid active material, are described as [33], [36], [48]:

(2) $$\begin{array}{c} \frac{\partial c_{m}}{\partial t}=-\nabla \cdot \overset{\to }{N_{m}}\\ \frac{\partial \rho_{m}}{\partial t}=-\nabla \cdot \overset{\to }{J_{m}} \end{array}$$

For a binary electrolyte, lithium ions, and electrons are the charged species that react at the particle-electrolyte interface. Here, $c_{m}$ is the lithium concentration in the *m*th medium with *m* denoting *e* for electrolyte and *s* for solid phases. $\overset{\to }{N_{i}}$ is the molar flux and $\rho_{m}$ is the charge density, $\overset{\to }{J_{m}}$ is the charge flux or the current density.

In the electrolyte phase, the lithium ion flux is driven by gradients in the electrochemical potential as given by Stefan-Maxwell's concentrated solution theory [32], [36], [59], [60]. The molar flux is due to diffusion and electromigration of lithium ions given as, $\overset{\to }{N_{e}}=-D_{e}^{\circ }\nabla c_{e}+\frac{t_{+}}{F}\overset{\to }{J_{e}}$, and the charge flux is driven by gradients in electrical potential as well as the diffusion of lithium ions which is described by Ohm's law as, $\overset{\to }{J_{e}}=-\sigma_{e}^{\circ }\nabla \phi_{e}+\sigma_{e}^{\circ }\frac{2RT(1-t_{+})}{F}\left[1+\frac{\partial \ln f_{\pm }}{\partial \ln\overset{˜}{c_{e}}}\right]\nabla \ln(\overset{˜}{c_{e}})$ [33], [36], [48], [60]. The term $\left[1+\frac{\partial \ln f_{\pm }}{\partial \ln\overset{˜}{c_{e}}}\right]$ is the thermodynamic factor [33], [61] and it is equal to one for an ideal solution.

In the solid phase, the molar flux is only due to diffusion, $\overset{\to }{N_{s}}=-D_{s}\nabla c_{s}$, and the charge flux or current density is due to the flow of electrons, given by Ohm's law, $\overset{\to }{J_{s}}=-\sigma_{s}^{\circ }\nabla \phi_{s}$, because the electromigration of lithium ions in the solid phase is negligible [33], [36], [48], [60].

Further, the lithium flux due to the charge transfer reaction at the particle-electrolyte interface, Equation (1) is a Neumann boundary condition at the particle surface, $\overset{ˆ}{n}$, *i.e.*, ${\nabla \cdot \overset{\to }{N_{s}}|}_{\overset{ˆ}{n}}=-j/F$ and ${\nabla \cdot \overset{\to }{N_{e}}|}_{\overset{ˆ}{n}}=j/F$, and ${\nabla \cdot \overset{\to }{J_{s}}|}_{\overset{ˆ}{n}}=-j$ and ${\nabla \cdot \overset{\to }{J_{e}}|}_{\overset{ˆ}{n}}=j$, since the total lithium mass and charge are conserved.

By using volume averaging on a representative volume element [48], [58], Equation set (2), results in:

(3) $$\begin{array}{c} \frac{\partial {\bar{c}}_{m}}{\partial t}=-\nabla \cdot \bar{\overset{\to }{N_{m}}}+{\bar{R}}_{c_{m}}\\ \frac{\partial {\bar{\rho }}_{m}}{\partial t}=-\nabla \cdot \bar{\overset{\to }{J_{m}}}+{\bar{R}}_{\rho_{m}} \end{array}$$

where, ${\bar{c}}_{m}$ and ${\bar{\rho }}_{m}$ are position-dependent volume averaged concentration and charge density, $\bar{\overset{\to }{N_{m}}}$ and $\bar{\overset{\to }{J_{m}}}$ are the volume averaged molar flux and current density, while ${\bar{R}}_{c_{m}}=aj/F$ and ${\bar{R}}_{\rho_{m}}=aj$.

#### 2.4.2 Mass transport in solid phase

Historically, the particle size distribution of active material is approximated as a statistically representative monodispersed distribution of spherical particles of radius, $r_{p}$, whose lithium mass transport is described by the diffusion equation in spherical coordinates [32], [39], [44], [62]:

(4) $$\begin{array}{c} \frac{\partial c_{s}}{\partial t}=-\nabla \cdot \overset{\to }{N_{s}}=\nabla \cdot \left(D_{s}\nabla c_{s}\right)\\ \;=\frac{1}{r^{2}}\frac{\partial }{\partial r}\left(r^{2}D_{s}\frac{\partial c_{s}}{\partial r}\right) \end{array}$$

where the Butler-Volmer relation, Equation (1), is imposed at the surface of the particle. The average lithium concentration in the particle corresponds to the electrode level concentration at the through-thickness location in the electrode.

#### 2.4.3 Mass transport in electrolyte phase

By starting from Equation (3), [32], [36], [44], [48]:

(5) $$\begin{array}{c} \frac{\partial \epsilon c_{e}}{\partial t}=-\nabla \cdot \bar{\overset{\to }{N_{e}}}+a\frac{j}{F}\\ \;=\nabla \cdot \left(\frac{D_{e}^{\circ }}{\tau }\nabla \epsilon c_{e}\right)-\nabla \cdot \left(\frac{t_{+}}{F}\bar{\overset{\to }{J_{e}}}\right)+a\frac{j}{F} \end{array}$$

Here, $\epsilon c_{e}$, is the average lithium concentration. Further, for constant porosity, constant lithium transference number, $t_{+}$, Equation (5) reduces to:

(6) $$\begin{array}{c} \epsilon \frac{\partial c_{e}}{\partial t}=\frac{\partial }{\partial x}\left(D_{e}^{\mathrm{eff}}\frac{\partial c_{e}}{\partial x}\right)+a\left(1-t_{+}\right)\frac{j}{F} \end{array}$$

The lithium ion concentration, $c_{e}$, and diffusion mass flux, $D_{\mathrm{eff}}^{e}\frac{\partial c_{e}}{\partial x}$, are continuous across the electrode-separator boundary, and requires a zero flux at both ends of the LIB.

#### 2.4.4 Charge transport in electrolyte phase

The volume averaged charge transport given by Equation (3) [32], [36], [44], [48]:

(7) $$\frac{\partial \epsilon \rho_{e}}{\partial t}=-\nabla \cdot \bar{\overset{\to }{J_{e}}}+aj$$

Thus, the porosity of the electrode, *ϵ*, is the fraction of volume occupied by the electrolyte phase, and $\epsilon \rho_{e}$, is the average electrolyte charge density. Similarly, the average charge density accumulation rate produced in the electrolyte phase at the active solid surface is, ${\bar{R}}_{\rho_{e}}=aj$.

In agreement with Doyle et al. [32], De Vidts et al. [48], and Smith et al. [36], the volume averaged Ohm's law in the electrolyte phase is: $\bar{\overset{\to }{J_{e}}}=-\frac{\sigma_{\circ }^{e}}{\tau }\nabla (\epsilon \phi_{e})+2\frac{(1-t_{+})RT}{F}\frac{\sigma_{\circ }^{e}}{\tau }\left[1+\frac{\partial \ln f_{\pm }}{\partial \ln\overset{˜}{c_{e}}}\right]\nabla (\epsilon \ln(\overset{˜}{c_{e}}))$ [36], [48], which leads to:

(8) $$\begin{array}{c} \frac{\partial \epsilon \rho_{e}}{\partial t}=\nabla \cdot \frac{\sigma_{\circ }^{e}}{\tau }\nabla (\epsilon \phi_{e})-\nabla \cdot \left(2\frac{(1-t_{+})RT}{F}\frac{\sigma_{\circ }^{e}}{\tau }\left[1+\frac{\partial \ln f_{\pm }}{\partial \ln\overset{˜}{c_{e}}}\right]\nabla (\epsilon \ln(\overset{˜}{c_{e}}))\right)+aj∼0 \end{array}$$

Further, due to the charge neutrality of the system, the net charge local charge accumulation rate is independent of time, $\frac{\partial \epsilon \rho_{e}}{\partial t}\approx 0$. For a constant, position-independent electrode porosity, the above equation is simplified in one dimension as [32], [36], [39], [44], [62]:

(9) $$\begin{array}{c} aj=-\frac{\partial }{\partial x}\left(\sigma_{e}^{\mathrm{eff}}\frac{\partial \phi_{e}}{\partial x}\right)+\frac{\partial }{\partial x}\left(\frac{2(1-t_{+})RT}{F}\sigma_{e}^{\mathrm{eff}}\left[1+\frac{\partial \ln f_{\pm }}{\partial \ln\overset{˜}{c_{e}}}\right]\frac{\partial \ln(\overset{˜}{c_{e}})}{\partial x}\right) \end{array}$$

The electric potential, $\phi_{e}$, and charge flux, $\sigma_{e}^{\mathrm{eff}}\frac{\partial \phi_{e}}{\partial x}$, are continuous across the electrode-separator interface and requires a zero charge flux at the ends of the LIB.

#### 2.4.5 Charge transport in solid phase

The volume averaged Ohm's law is: $\bar{\overset{\to }{J_{s}}}=-\frac{\sigma_{s}^{\circ }}{\tau }\nabla ((1-\epsilon )\phi_{s})$, in agreement with [36], [48]. Equation (3), along with the charge continuity equation, at steady state, is:

(10) $$\begin{array}{c} \frac{\partial (1-\epsilon )\rho_{s}}{\partial t}=-\nabla \cdot \bar{\overset{\to }{J_{s}}}-aj∼0\\ \;=\nabla \cdot \left(\frac{\sigma_{s}^{\circ }}{\tau }\nabla ((1-\epsilon )\phi_{s})\right)-aj \end{array}$$

The average charge density consumed per unit time in the solid phase is, $R_{\rho_{e}}=-aj$, which is equal and opposite to the charge flux contribution entering the electrolyte phase [32], [36], [39], [44], [62]. Thus, for a 1D system:

(11) $$\begin{array}{c} -aj=-\frac{\partial }{\partial x}\left(\sigma_{s}^{\mathrm{eff}}\frac{\partial \phi_{s}}{\partial x}\right) \end{array}$$

A Neumann boundary condition is applied at the ends of the electrode, such that the charge flux is zero at the electrode-separator boundary, while the charge flux at the ends of the LIB is equal to the applied current density $\overset{\to }{I}=I_{\mathrm{app}}\overset{ˆ}{n}$, which is constant during galvanostatic charge-discharge of the cell. By combining Equations (7) and (10), and using charge conservation, $\bar{\overset{\to }{J_{e}}}+\bar{\overset{\to }{J_{s}}}=I_{\mathrm{app}}\overset{ˆ}{n}$.

## 3 Methods

### 3.1 Generalities

A LiMn_2_O_4_-graphite cell with plasticized electrolyte (2M $\mathrm{LiP}F_{6}$ in 1:2 EC:DMC) as reported by Doyle et al. [46] was considered to analyze the performance of three open source porous electrode theory codes: dualfoil v5.2 [43], MPET v0.1.6 [36], [63] and LIONSIMBA v2.1 [39], [64]. The anode, separator, and cathode layers are discretized, and Equations (1), (6), (9), and (11) are solved in each layer, while Equation (4) is solved for a representative spherical particle present at each node of the mesh locations in anode and cathode. The average concentration in the particle is considered as the solid phase concentration in the electrode at that mesh location.

The cell parameters used for the simulations, see Table 2, correspond to those reported by Doyle et al. [46], in order to ensure a fair comparison across the different PET implementations. See supplemental for the utilized input files. In the current setup, the plasticized gel electrolyte is composed of polymer volume fraction, $\epsilon^{p}$ and the liquid electrolyte volume fraction $\epsilon^{e}$, thus the porosity considered while solving the transport equations in the electrolyte is $\epsilon =\epsilon^{e}+\epsilon^{p}$. While the solid phase occupies the remaining volume fraction, 1−ϵ, it contains conductive filler that has a volume fraction of $\epsilon^{f}$, so the reactive area density of the electrode is $a=\frac{3(1-\epsilon^{e}-\epsilon^{p}-\epsilon^{f})}{r_{p}}$.

**Table 2:** Summary of the LiMn_2_O_4_-graphite cell parameters used for simulations [46].

Parameters	Symbol	Cathode	Separator and Electrolyte	Anode	Units
material		LiMn_2_O_4_	separator: plasticized gel electrolyte 2M LiPF_6_ in 1:2 ratio mixture of EC/DMC	LiC_6_	–
thickness	*L*_*i*_	174	52	100	μm
density	*ρ*_*i*_	4.14	polymer phase: 1.324 liquid phase: 1.780	1.9	g/cm^3^
particle radius	*r*_*p*,*i*_	8.5	–	12.5	μm
particle shape factor	*S*_*i*_	3	–	3	–
volume fraction of electrolyte	$\epsilon_{i}^{e}$	0.444	0.724	0.357	–
volume fraction of polymer phase	$\epsilon_{i}^{p}$	0.186	0.276	0.146	–
volume fraction of filler	$\epsilon_{i}^{f}$	0.073	–	0.026	–
bruggeman exponent	*b*_*i*_	0.5	2.3	0.5	–
diffusion coefficient of Li in solid	$D_{i}^{s}$	1 × 10^−9^	–	0.39 × 10^−9^	cm^2^/s
diffusion coefficient of Li in electrolyte	$D_{\circ ,i}^{e}$	7.5 × 10^−7^	7.5 × 10^−7^	7.5 × 10^−7^	cm^2^/s
initial concentration of Li in solid	$c_{s,i}^{\circ }$	3.9	–	14.87	mol/dm^3^
initial salt concentration in electrolyte	$c_{e,i}^{\circ }$	2.0	2.0	2.0	mol/dm^3^
maximum concentration in solid	$c_{s,i}^{\max}$	22.86	–	26.39	mol/dm^3^
initial exchange current density	*i*_0,*i*_	0.08	–	0.11	mA/cm^2^
conductivity of solid matrix	$\sigma_{\mathrm{eff},i}^{s}$	0.038	–	1.0	S/cm
absolute temperature	*T*	298.15	298.15	298.15	K
number of discretization points along the thickness	*n*_*i*_	87	26	50	-
number of discretization points in the electrode particle	*n*_*p*,*i*_	20	-	20	-

All three codes require a large set of input parameters, and some input parameter descriptions and definitions were inconsistent across the codes. For example, dualfoil and LIONSIMBA use the reaction rate constant *k* as an input parameter, instead MPET uses a non-dimensionalized initial exchange current density $i_{0}$ as an input parameter. The solubility limit or maximum concentration of lithium in solid phase $c_{s}^{\max}$ is an input parameter for dualfoil and LIONSIMBA, while MPET uses total lithium site density estimated as $N_{A}c_{s}^{\max}$, where $N_{A}$ is the Avogadro constant. LIONSIMBA uses the reactive area density as an input parameter, while dualfoil and MPET calculate the parameter using the shape factor, porosity, and radius of the representative particle. Thus, input parameter descriptions were identified and matched to the required cell parameter values along with the numerical tolerances of 10^−8^, to ensure the simulation setup was the same for the three codes. The differences in the numerical approach for these codes are listed in Table 3. Section 5 summarizes the differences in the electrochemical response, and provides a context to quantify the differences on physical, though-thickness response, and their implications on the location, time, and extent of the resultant degradation kinetics that would develop as a result of selecting any of these models.

**Table 3:** Summary of differences in the numerical approach.

Method or feature	dualfoil [32]	MPET [36]	LIONSIMBA [39]
programming language	Fortran (f77)	Python 3.6	MATLAB
solver approach	finite difference	finite volume	finite volume
numerical solver	matrix solver (BAND) [43]	DAE Tools v1.8 [63]	Sundials v2.6.2 (IDA) [64]
upscaling solid phase diffusion	superposition integral [32]	average particle concentration	average particle concentration
reference electrode (for potential)	cathode (*x* = *L*)	pure lithium Li/Li^+^	anode (*x* = 0)

All three codes, Dualfoil, MPET and LIONSIMBA were installed and run on a Ubuntu 14.04.5 OS, utilizing a single core out of 32 cores available in an Intel(R) Xeon(R) CPU E5-2698 v3 @ 2.30 GHz, RAM of 16 GB out of 128 GB.

### 3.2 Benchmarking tools

A python wrapper was implemented, one for dualfoil and another for MPET to automate the input of material parameters into the different codes. The developed user interface supports (a) a graphical mode to interactively launch simulations, and (b) a command line mode to script the commands and to launch batches of simulations into a computer server. dualfoil.py, user interface for dualfoil was developed by Robinson and García [65], and it was further improved for the current study [66]. The developed user interface for MPET v0.1.6, mpetUI [67], provides equivalent functionality as the dualfoil interface. The graphical mode of the user interface was used for parameter input and to launch simulations of cell discharge at different C-rates, while the command line mode was used to setup 700 charge-discharge cycles simulation for performance analysis. Although no user interface was developed for LIONSIMBA simulations, the current density function file was modified to represent the cycling currents to simulate 700 cycles of charge-discharge.

Further, an input file converter, bat2bat [68], was developed to convert input files between source and target codes with a 50% performance.

### 3.3 Dualfoil overview

Dualfoil uses Duhamel's superposition integral to approximate the solution for solid phase concentration on electrode particles, Equation (4) [21], [44]. Dualfoil implements a second-order finite difference in space and Crank-Nicolson implicit scheme for time derivatives to discretize Equations (6), (9), and (11) in Fortran [44]. The matrix solver BAND [33], [43] was used to solve the system of algebraic equations. Dualfoil uses the cathode (x=L) as the reference for electrolyte potential calculations [44]. The relative and absolute tolerance values of 10^−8^ were utilized during the simulation.

### 3.4 LIONSIMBA overview

LIONSIMBA utilizes a finite volume approach and the method of lines to implement Equations (4), (6), (9), and (11), into differential algebraic equations, DAEs, by using a second order finite differences in space [39]. The equations are iteratively solved using IDA solver from Sundials v.2.6.2 [69] on MATLAB [39], [64]. The diffusion equation on the particle scale, Equation (4) is solved, and the average particle concentration is reported as the solid phase concentration at the particle location in the electrode. LIONSIMBA uses the anode (x=0) as the reference electrode for electrolyte potential calculations [39]. The relative and absolute tolerance values of 10^−8^ were utilized during the simulation.

### 3.5 MPET overview

MPET utilizes a finite volume approach similar to LIONSIMBA [36]. The IDAS solver from SUNDIALS integration suite [69] was used, with a fifth-order adaptive backward difference formula time stepper [36], [63]. The average particle concentration obtained after solving the particle scale diffusion equation is reported as the solid phase concentration at the through-thickness location in the electrode. MPET uses pure lithium (Li/Li^+^) as the reference electrode for electrolyte potential calculations [36]. The relative and absolute tolerance values of 10^−8^ were utilized during the simulation.

## 4 Performance analysis

**Figure 2:**
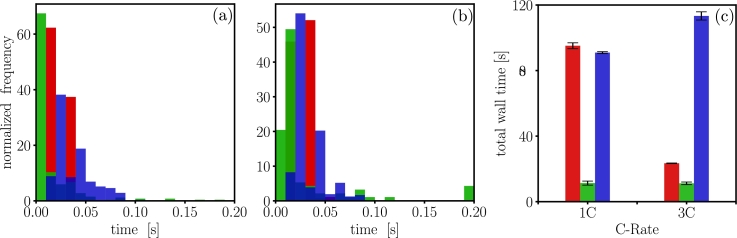
Time histograms highlighting average wall time per iteration and total wall time of simulations for 1C and 3C discharge, for dualfoil , MPET  and LIONSIMBA . Insets (a) correspond to single time step wall time histogram for 1C discharge. Insets (b) correspond to single time step wall time histogram for 3C discharge. The histograms represent the frequency of simulation wall time per iteration time step, normalized with the total number of iteration time steps. The position of the peak value in the histogram qualitatively shows which model runs faster. Inset (c), shows the total simulation wall time, suggests that MPET runs faster compared to the other two codes.

The computational speeds of the three open source PET models are benchmarked in Fig. 2. The adaptive time-stepping algorithm implemented in all three models allows for a varied number of time steps to simulate full discharge. The performance comparison for all three codes is summarized in Table 4. The average simulation time for dualfoil is short because it is a Fortran based code, but it takes more computation time due to Duhamel's superposition approximation [21]. The total simulation wall time for MPET is much lower compared to the other two models due to the higher-order adaptive numerical time stepping scheme used. The total simulation wall time for LIONSIMBA is affected by MATLAB being an interpreted language.

**Table 4:** Performance comparison of three PET models for simulating 1C and 3C discharge.

	1C-rate			3C-rate		
Model	dualfoil	MPET	LIONSIMBA	dualfoil	MPET	LIONSIMBA
total wall time (s)	95.13 ± 1.8	11.32 ± 1.22	90.95 ± 0.58	23.42 ± 0.16	11.17 ± 0.8	113.32 ± 2.5
number of time steps	3408 ± 10	253 ± 6	1433 ± 2	894 ± 10	94 ± 5	2392 ± 3
average wall time per time step solved (ms)	27.94 ± 6.3	33.64 ± 18.2	63.92 ± 28.2	26.15 ± 6.95	72.22 ± 52.1	47.37 ± 15.32
initialization time (s)	0.6 ± 0.02	2.26 ± 0.03	9.98 ± 1.7	0.083 ± 0.02	3.85 ± 0.7	10.16 ± 1.3
time to write to file (s)	1.21 ± 0.08	1.02 ± 0.63	0.10 ± 0.005	0.077 ± 0.01	1.10 ± 0.4	0.12 ± 0.002

**Figure 3:**
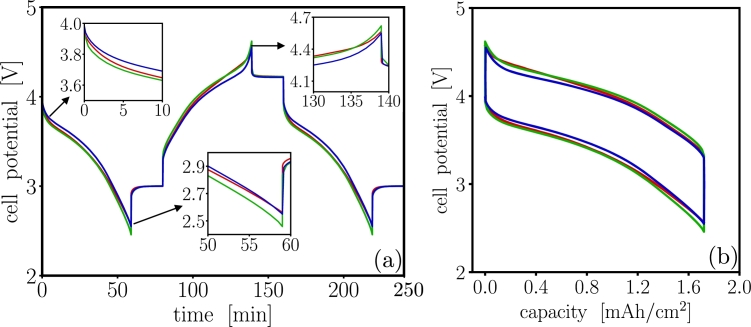
Cycling performance of the three codes, dualfoil , MPET  and LIONSIMBA . Each cycle consisted discharging for 59 min at 17.5 A/m^2^ followed by resting for 21 min at 0.0 A/m^2^, then charging for 59 min at 17.5 A/m^2^ followed by resting for 21 min at 0.0 A/m^2^. Inset (a) shows cell potential *vs* time for one representative cycle. Inset (b) shows cell voltage *vs* capacity. Simulations demonstrate that the drift of charge capacity gain/loss is ∼10^−6^ mAh per cycle.

The cycling performance of the three codes is summarized in Fig. 3 for 700 cycles at 1C. A resting time of 20 minutes between each charge or discharge event was imposed to allow the cell to reach equilibrium after charge and discharge. Without any loss of generality, we have arbitrarily set the cutoff voltage to be 4.7 V and 2 V. In an experimental setup these would be specified to reflect the operating and safety voltage regimes of the device. Although the analytical equation of mass conservation should conserve mass, the numerical implementation of a mass conservation equation may not conserve mass [70], and should always be tested. Here, results show the change in charge capacity (the numerical charge capacity loss) is $∼10^{-6}$ mAh per cycle, therefore all of the three analyzed PET models show that the mass of cyclable lithium in the cell is conserved during the charge-discharge cycling simulations, which is a physically required preamble to perform any degradation simulations.

## 5 Results and discussion

**Figure 4:**
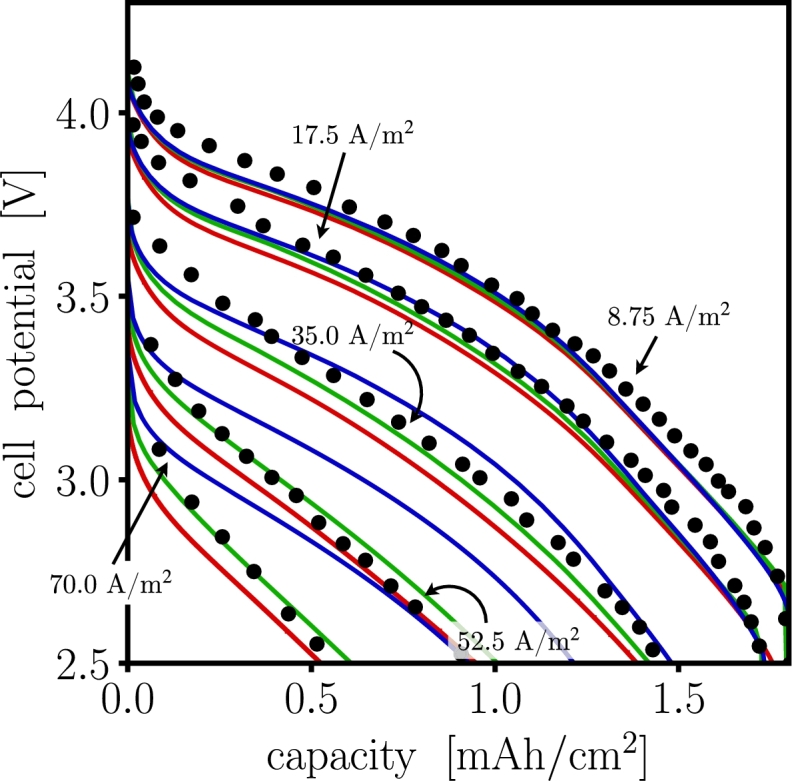
Cell voltage – charge capacity comparison for dualfoil  MPET  and LIONSIMBA  the experimental data •, at selected C-rates. Used model parameters correspond to those reported by Doyle et al. [46]. 1C corresponds to a current density of 17.5 A/m^2^. The cell potential for Dualfoil and MPET show a deviation with less than 5% for all C-rates, while LIONSIMBA deviates ∼10% for C-rates above 2C.

Fig. 4 shows the predicted voltage *vs* charge capacity compared against the corresponding experimental response as reported by Doyle et al. [46]. The reaction rate parameters of, $k_{a}=1.947\times 10^{-11}\;m^{2.5}\;\mathrm{mo}l^{-0.5}\;s^{-1}$ for the anode and $k_{c}=2.156\times 10^{-11}\;m^{2.5}\;\mathrm{mo}l^{-0.5}\;s^{-1}$ for the cathode, correspond to the initial exchange current density, $i_{0,i}$, values listed in Table 2. These parameters along with other parameters listed in Table 2 were utilized in all three PET implementations. Differences in root mean squared (RMS) deviations are observed in each software. For low C-rates (≤ 1C), they all show nearly identical behavior. For high C-rates (≥ 2C), both dualfoil and MPET underestimate the voltage response. In all cases, the increase in the difference of behavior is a result of failure to satisfy the homogenization approximation assumption [25]. Here, the local microstructural heterogeneities become more significant and their impact cannot be captured efficiently in each of the corresponding numerical implementations.

**Table 5:** Deviation of simulations from the experimental data, in Fig. 4, are calculated as RMS %.

C-rate	C/2	1C	2C	3C	4C
dualfoil	2.224	2.261	3.409	3.558	2.799
MPET	3.393	3.548	2.598	4.121	2.680
LIONSIMBA	2.572	2.072	1.331	10.404	11.934

**Figure 5:**
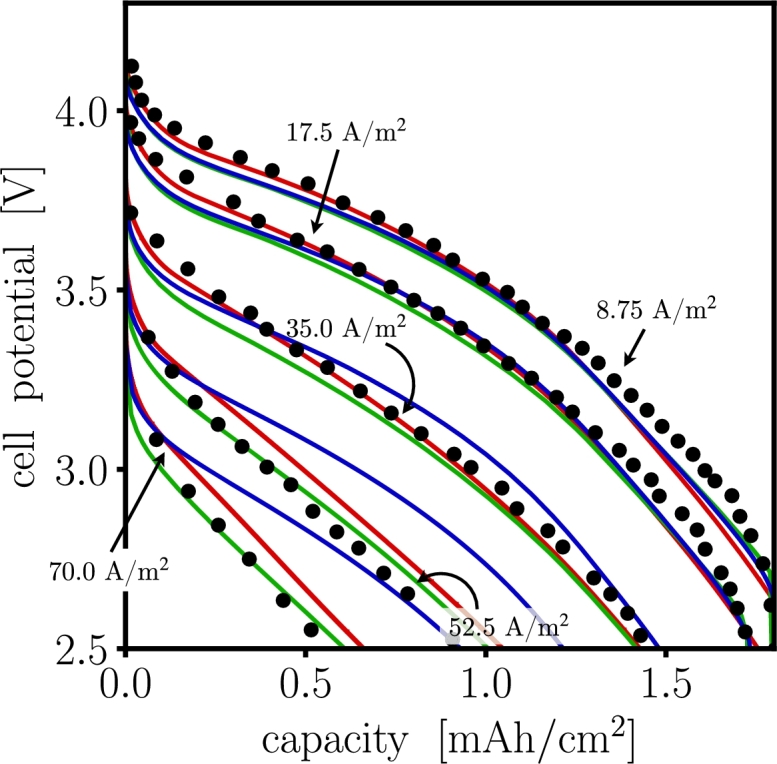
Attempt to improve the fit of cell voltage *vs* charge capacity for dualfoil  MPET  and LIONSIMBA  the experimental data •, from Doyle et al. [46] at different C-rates. The initial exchange current densities were adjusted to improve the comparisons with experimental data. While only one unique true set of physical parameters exists that will deliver the true solution, an infinite number of combinations exist that would fit the model to the experimental voltage response.

An attempt to improve on the cell voltage *vs* charge capacity for the experiments was obtained by adjusting the reaction rate parameters in dualfoil and LIONSIMBA to be $k_{a}=1.38\times 10^{-5}\;m^{2.5}\mathrm{mo}l^{-0.5}s^{-1}$ on the anode and $k_{c}=6.4\times 10^{-5}\;m^{2.5}\mathrm{mo}l^{-0.5}s^{-1}$ on the cathode, so the initial exchange current density parameter in MPET was adjusted to $i_{0,a}=1.5683\;\mathrm{mA}/cm^{2}$ and $i_{0,c}=1.5042\;\mathrm{mA}/cm^{2}$ for anode and cathode. The rest of the parameters in Table 2 remained the same. Fig. 5 shows the comparison of the models, the RMS deviation for MPET and dualfoil are less than 3.5% for all C-rates, while LIONSIMBA shows a deviation of about 9% for C-rates above 3C. Although the macroscopic response of the cell is explained with only one unique set of physical parameters, simulations suggest that many parameter combinations exist that reproduce the same macroscopic behavior.

**Figure 6:**
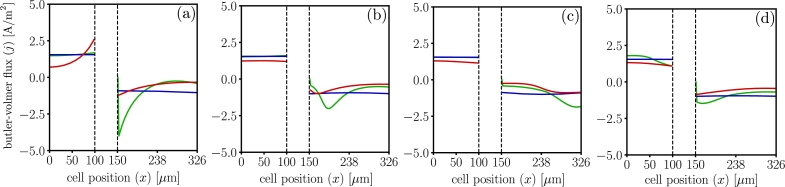
Butler-Volmer flux (*j*), profiles for dualfoil  MPET  LIONSIMBA  1C-rate, inset (a) corresponds to t=0 minutes (b) t=20 minutes (c) t=40 minutes and (d) t=59 minutes. Although the macroscopic cell potentials match and the qualitative trends of the Butler-Volmer flux profiles agree, the numerical differences and assumptions regarding homogenization approximation affect the electrochemical profiles.

Fig. 6 shows the comparison of the through-thickness Butler-Volmer flux, *j*, calculated by all three models at different instances during battery discharge, lithium intercalates in the cathode and deintercalates in the anode, which results in a positive source of charge in the anode and a negative source of charge in the cathode. The initial Butler-Volmer flux is different across all the models, as seen in Fig. 6(a), because dualfoil uses Duhamel's superposition integral approximation for the electrode particle surface concentration, while LIONSIMBA and MPET use the particle surface concentration after numerically solving Equation (4). The differences in the Bulter-Volmer response by each model observed suggest: 1) dualfoil shows large, localized deintercalation rates in the anode section abutting the separator at the beginning of discharge, 2) MPET shows a reaction zone that propagates from the separator-cathode interface to the back-contact, 3) LIONSIMBA shows the intercalation reaction occurs homogeneously in both electrodes. Consequently, different conclusions are drawn by all three models.

**Figure 7:**
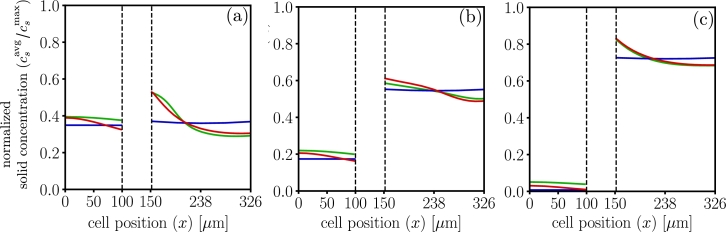
Normalized solid phase concentration profiles, for dualfoil , MPET  and LIONSIMBA  at 1C-rate inset (a) corresponds to t=20 minutes (b) t=40 minutes and (c) t=59 minutes. Results demonstrate that spatial localization of intercalation reaction induces local accumulation in the particles of active material.

Fig. 7 shows the corresponding normalized lithium concentration in the solid phase for all three models at selected instants during discharge. At the beginning of the discharge, the normalized average concentration in the anode is $\frac{c_{s}^{\mathrm{avg}}}{c_{s}^{\max}}=0.5635$ and the cathode is $\frac{c_{s}^{\mathrm{avg}}}{c_{s}^{\max}}=0.1705$. The differences in the concentration profiles are a result of the differences in the Butler-Volmer flux for each model, thus the large gradients can be physically interpreted in different ways, thus suggesting accumulation and the associated degradation mechanisms that may ensue to be more likely in some models, but not in others [71], [72], [73].

**Figure 8:**
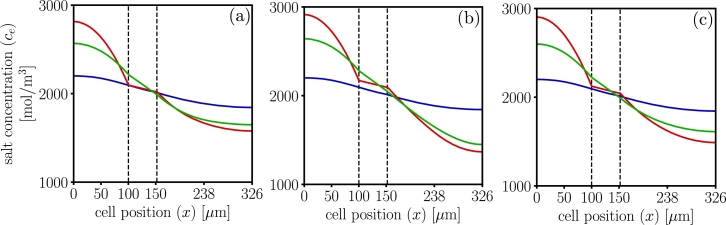
Salt concentration profiles in electrolyte phase for dualfoil , MPET  and LIONSIMBA  at 1C-rate. Insets (a) corresponds to t=20 minutes (b) t=40 minutes and (c) t=59 minutes. Results show that dualfoil predicts the largest accumulation and depletion of salt concentration. The salt concentration gradient in the separator is different for MPET compared to dualfoil and LIONSIMBA, due to a mismatch in the porosity corrections used.

The corresponding comparison of the salt concentration profiles for all three models for 1C discharge is shown in Fig. 8. Dualfoil shows largest salt accumulation and depletion regions, suggesting that salt precipitation is more likely. The concentration gradient in the separator region is the same for Dualfoil and LIONSIMBA.

**Figure 9:**
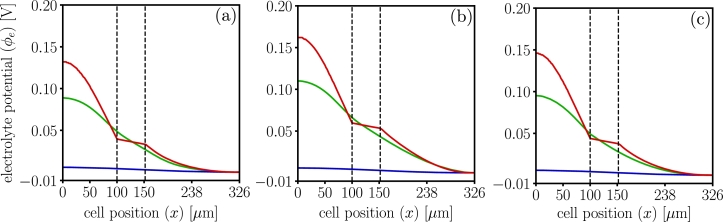
Electrolyte phase potential profiles for dualfoil , MPET  and LIONSIMBA  at 1C-rate. Insets (a) corresponds to t=20 minutes (b) t=40 minutes and (c) t=59 minutes. The three models use different reference electrodes: dualfoil used cathode, MPET used pure lithium and LIONSIMBA used anode. Here, the cathode was reset as the reference phase for ease in the comparison.

Finally, Fig. 9 shows the potential of the electrolyte phase with the cathode end as a reference since the three models use different reference electrodes for potential calculations. Dualfoil shows larger electrolyte potential in the anode since the salt concentration predicted is higher than other models.

**Figure 10:**
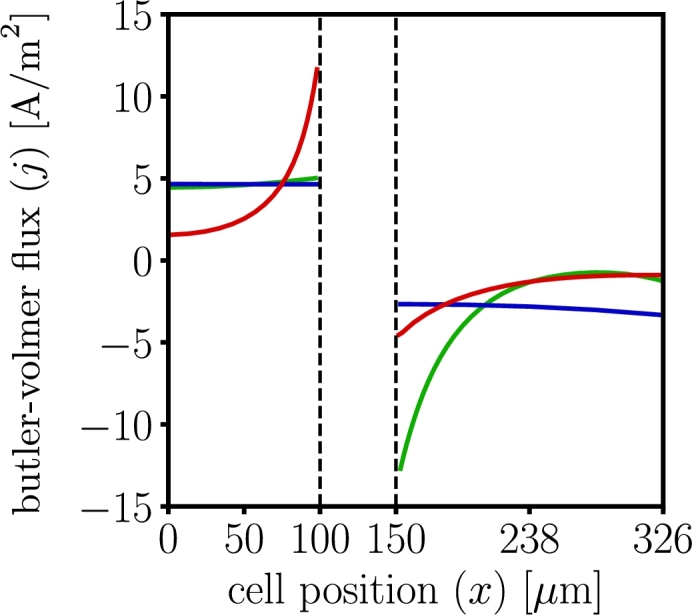
Butler-Volmer flux for dualfoil , MPET  and LIONSIMBA  for 3C-rate discharge at t=0 minutes. The results show a numerical disagreement in the local flux prediction due to differences in the implementation of the homogenization techniques, which lead to differences in other electrochemical profiles at 3C.

The effects observed in Figure 6, Figure 7, Figure 8, Figure 9 are exacerbated for high C-rates. Specifically, Fig. 10 shows that for a 3C discharge, the differences are higher particularly close to the separator-electrode layer interface, suggesting that localized side reactions, such as chemomechanically-induced particle fracture, solid electrolyte interphase growth, and dendrite formation, are more likely in some models [25], [71].

**Figure 11:**
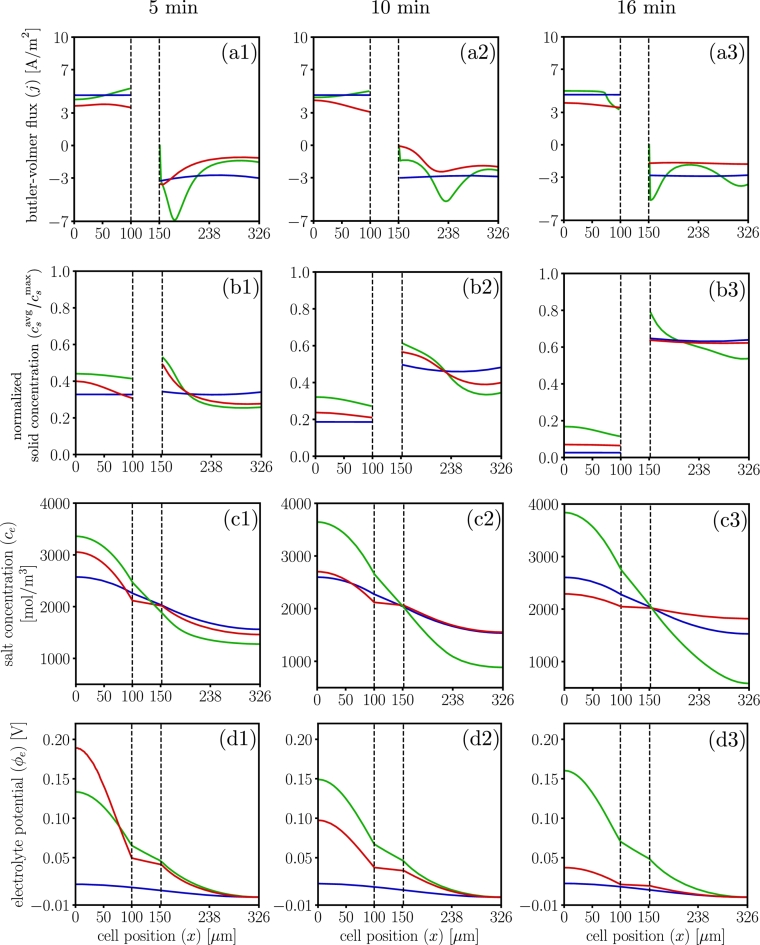
Electrochemical profiles at 3C-rate, for dualfoil , MPET  and LIONSIMBA . Insets (a)1 to (a)3 show the Butler-Volmer flux profiles at t=5,t=10, and t=16 minutes. Insets (b)1 to (b)3 show the normalized solid surface concentration profiles at t=5, t=10, and t=16 minutes. Insets (c)1 to (c)3 show the salt concentration profiles at t=5, t=10, and t=16 minutes. Insets (d)1 to (d)3 show the electrolyte potential profiles at t=5, t=10, and t=16 minutes.

Fig. 11 compares the electrochemical response for a 3C discharge at selected instants. The differences in Buther-Volmer reaction distribution lead to differences in the concentration profiles and the electrolyte potential. In particular, while LIONSIMBA suggests no reaction zone, dualfoil suggests one reaction zone in the cathode, and MPET suggests that a reaction zone in both the anode and cathode. This greatly impacts the local electrochemical lithium transport, see Fig. 11(a).

The appearance of each reaction zone is directly correlated to the localized accumulation of lithium in the solid phase, see Fig. 11(b), having long term implications on the energy density and charge capacity predictions, particularly at the end of the discharge cycle. Furthermore, MPET results suggests the greatest possibility of salt precipitation will be in the anode side, and lithium depletion in the cathode, while dualfoil suggests that is likely at the beginning of the discharge, and LIONSIMBA in the middle of the sequence, see Fig. 11(c). Finally, dualfoil results suggests that the overpotential-induced degradation mechanisms are more likely on the back contact region of the anode at the beginning of the discharge, while MPET suggests it is more likely as the discharge process increases, see Fig. 11(d).

## 6 Summary and conclusions

The performance of three PET codes dualfoil, MPET, and LIONSIMBA was compared.^2^ Benchmarking tools were developed in order to facilitate easier simulation across the codes, and the framework developed herein is applicable to other types of lithium ion battery electrochemical models, such as the single particle models. The simulated discharge voltage curves of a LiMn_2_O_4_-graphite cell were close to the experiments, for C-rate below 2C the RMS deviations are less than 5%. For, higher C-rates the model predictions deviated as the homogenization approximation used by the models is insufficient to efficiently capture the microstructural heterogeneities that become important at high C-rates. Even though the predicted macroscopic behavior from all three codes is similar within 5% RMS error at 1C-rate discharge there are great differences in the predicted electrochemical profiles, and these differences are further enhanced at 3C-rate discharge. Therefore, the PET codes need to be benchmarked with the experimentally observed macroscopic behavior as well as the electrochemical profiles to provide physically accurate predictions. The large differences in the through-thickness behavior among these models imply the possibility of large differences on the predicted degradation mechanisms (if iimplemented on top of each framework), thus making the implementation of benchmarks and tests that allow these models to provide reliable results, a requirement for valid calculations.

A comparison of computational performance showed that MPET runs ∼10 times faster compared to the other two codes for the same numerical setup, due to the solver and adaptive time stepping schemes implemented, and benefits being a python based code. LIONSIMBA is limited by MATLAB being an interpreted language and takes significant initialization time to load the solver and other dependencies. The speed of Dualfoil, a Fortran based code, is a result of the implemented numerical scheme. The long-term charge-discharge cycling performance of the three codes was evaluated to show that there is a net loss of ∼10^−6^ charge capacity per cycle, which implies the simulations show the lithium is conserved in te abscence of degradation degradation mechanisms. Overall, this analysis suggests that PET models should benchmark the electrochemical behavior along with the macroscopic behavior. Also, a consistent nomenclature for the parameter definitions across all models would be useful for any user to easily understand and launch PET simulations irrespective of the code. The performed analysis highlights that the emergent differences in the localization of the electrochemical fields, including differences on the solid and salt concentrations, electrolyte potential and Butler-Volmer fluxes, bring up the important question on whether or not a model prediction is in agreement with the experiment. In this context, the researcher should compare the model results against experiments to accurately assess whether the calculation is representative of reality and arrive to their own scientific conclusions.

## CRediT authorship contribution statement

**Surya Mitra Ayalasomayajula:** Writing – review & editing, Writing – original draft, Validation, Software, Investigation, Formal analysis. **Daniel Cogswell:** Writing – review & editing, Formal analysis. **Debbie Zhuang:** Writing – review & editing, Investigation. **R. Edwin García:** Writing – review & editing, Writing – original draft, Supervision, Resources, Project administration, Methodology, Funding acquisition, Conceptualization.

## Declaration of Competing Interest

The authors declare that they have no known competing financial interests or personal relationships that could have appeared to influence the work reported in this paper.

## Appendix A Supplementary material

MMC Example input files used for the benchmarking analysis of Dualfoil, MPET and LIONSIMBA. MMC

## Footnotes

1 While the parameters utilized in this model are not necessarily representative of a modern LMO cell, they provide a common framework to develop a consistent understanding on the reaches and limitations of existing PET codes.

2 At the time of performing the benchmarking analysis shown herein, emerging PET codes such as pybamm [74] and petlion [75] were not known by the coauthors. Their emergence only stresses further the importance of establishing standards and tests across both public and commercial platforms. Nevertheless, the principles proposed in the paper are readily applicable to emerging open source PET softwares, without bias.
